# Acting Emotions: a comprehensive dataset of elicited emotions

**DOI:** 10.1038/s41597-024-02957-2

**Published:** 2024-01-31

**Authors:** Luís Aly, Leonor Godinho, Patricia Bota, Gilberto Bernardes, Hugo Plácido da Silva

**Affiliations:** 1 Faculty of Engineering, Department of Informatics Engineering, University of Porto, Porto, 4200-465 Portugal; 2 INESC-TEC, Telecommunications and Multimedia, Porto, 4200-465 Portugal; 3 Instituto de Telecomunicações, Instituto Superior Técnico, Department of Bioengineering, Lisbon, 1049-001 Portugal

**Keywords:** Engineering, Electrical and electronic engineering

## Abstract

Emotions encompass physiological systems that can be assessed through biosignals like electromyography and electrocardiography. Prior investigations in emotion recognition have primarily focused on general population samples, overlooking the specific context of theatre actors who possess exceptional abilities in conveying emotions to an audience, namely *acting emotions*. We conducted a study involving 11 professional actors to collect physiological data for *acting emotions* to investigate the correlation between biosignals and emotion expression. Our contribution is the DECEiVeR (**D**atas**E**t a**C**ting **E**mot**i**ons **V**alenc**e** a**R**ousal) dataset, a comprehensive collection of various physiological recordings meticulously curated to facilitate the recognition of a set of five emotions. Moreover, we conduct a preliminary analysis on modeling the recognition of acting emotions from raw, low- and mid-level temporal and spectral data and the reliability of physiological data across time. Our dataset aims to leverage a deeper understanding of the intricate interplay between biosignals and emotional expression. It provides valuable insights into acting emotion recognition and affective computing by exposing the degree to which biosignals capture emotions elicited from inner stimuli.

## Background & Summary

Researchers from multiple disciplines, such as psychology, cognition, and computing science, have demonstrated significant interest in the computer-based recognition of emotions, leading to affective computing as a field within computer science focused on enabling computers to detect and respond to human emotions^1^. Affective computing fosters harmonious human-computer interaction by facilitating computers’ understanding and appropriate response to human emotions^2^. Efforts in affective computing are directed towards refining computers’ ability to perceive and interpret human emotional states accurately, ultimately aiming to enhance the overall quality of user interactions and experiences in various domains such as healthcare, education, or entertainment.

Researchers commonly regard emotions as psychological states that find expression through facial expressions and can be discerned using computer vision techniques^3,4^. While facial recognition effectively identifies facial emotional expressions such as anger, disgust, and surprise, physiological sensors offer a distinct avenue for examining an individual’s emotional state^5^. Physiological sensors measure physiological activities (e.g., the electrical potential of neural impulses), including the heart, skin, and brain^6,7^, and can detect subtle changes in emotional states that are not apparent through facial expressions alone. Physiological sensors capture internal physiological responses related to the autonomic nervous and limbic systems, which individuals do not explicitly control. Recent research has explored how indicators like heart rate correlate with emotional expressions^8,9^, facial muscle activation^10,11^, or electrodermal activity^12^. In the study by Sharma and colleagues^13^, physiological signals were synchronized with real-time continuous annotation of emotions involving multiple participants watching various videos. However, the extent of agreement between subjective and physiological emotional responses over time, particularly regarding temporal dynamics and the degree to which these emotional portrayals resemble spontaneous emotional expressions, remain subjects of ongoing debate^14^.

The studies mentioned in the literature rely on five types of stimuli applied to one or more individuals, as described in^15^: (i) performing specific tasks, such as driving; (ii) exposure to externally driven stimuli, such as watching a movie; (iii) leader-follower interactions, as observed during a meeting; (iv) dynamic interactions, such as engaging in a conversation; and finally, (v) cooperative tasks. However, to the best of our knowledge, there needs to be more research that focuses on internally driven stimuli that arise from within an individual’s body rather than being externally perceived. These internal processes generate stimuli that can significantly influence an individual’s emotions or physiological responses.

Despite the growing interest in affective computing, most studies have primarily focused on recognizing emotions within general population samples. More attention should be given to investigating creative individuals’ emotional experiences and non-verbal expressions, particularly among theatre actors. As an art form, theatre provides an analytical metaphor that allows for the observation and exploration of elements from everyday life^16^. Theatre actors are of particular psychological interest in affective computing, not only because they receive approval from audiences but also due to their proficiency in controlling expressive behavior and their expertise in non-verbal emotional communication^17^. An exception to this research gap is the work by Zhang and colleagues^18^, which presented a dataset of actors’ kinematics in expressing six basic emotions.

To further investigate elicited emotions, i.e., internally driven emotions, we conducted a study involving 11 actors tasked with eliciting five emotions: neutral, calm, tiredness, tension, and excitement. For our study, we adopted the working definition of *acting emotions* as referring to the intentional elicitation of specific emotional states by the actors solely through internal stimuli. During the experiment, we collected various physiological measures, including electrocardiography (ECG), electrodermal activity (EDA), respiration (PZT), electromyography (EMG), and motion data. Data were compiled into a comprehensive and systematic dataset named DECEiVeR^19^, encompassing approximately seven hours of continuous recordings. Furthermore, the dataset incorporates the annotation data previously utilized in a publication that introduced and validated our approach to emotion annotation^20^. Additionally, our experiment protocol details a novel approach to emotion recognition. By eliciting emotions without any external input, participants reveal new insights invaluable to the field. This method allows for a more authentic and spontaneous expression of emotions, diverging from traditional emotion elicitation techniques. Our findings suggest that actors, in their pursuit of emotional authenticity, often employ creative strategies to convey emotions that may not align with conventional expressions. For instance, one participant (actor) noted that their portrayal of anger intentionally deceives, i.e., circumvents the obvious, offering the audience a fresh perspective on emotional expression.

## Methods

### Ethics Statement

The study received retrospective approval from the Data Protection Officer of The University of Porto, Portugal, under approval number 11094042/2023. The Officer evaluated and concluded that the risks to the rights and freedoms of participants contributing to this dataset were very low. This assessment was based on several factors: participants voluntarily engaged in the study after making an informed decision, and the data is considered anonymous when considering reasonable means (human, technological, temporal and financial) that could potentially be used for personal identification. The dataset comprises exclusively physiological signal data, each linked to individual participants through unique identification numbers assigned for the study. These numbers are not connected to the participants’ identities, ensuring the data’s anonymity.

Informed consent for data sharing and participation was obtained from all participants. They received detailed information about the study’s objectives, the methods used for data collection, and the measures taken to maintain confidentiality. Every participant was fully apprised of the nature of the research and how the collected data would be used, ensuring their participation was informed and voluntary. The study did not offer any financial incentives for participation.

### Participants

Eleven Portuguese professional actors (five females and six males with a mean age of 36 and a standard deviation of 10.82 years) participated in this study. Participants were recruited through an open call directed at professional theatre companies and schools. All participants reported no neurological or psychiatric problems.

It is important to note that our study focuses on a specialized population segment, specifically within the context of the Portuguese acting profession. In Portugal, the acting profession is relatively rare, so a sample size of 11 participants reflects this specialized group. Our study offers valuable insights specific to this context, contributing to the broader understanding of emotional expression in professional acting. The participants vary in age, and we have maintained a gender balance, adding diversity to our study. While we acknowledge the limitations regarding broader generalizability, we believe our findings are significant for understanding emotional elicitation in this specific professional group.

### Experiment design

In defining emotional experience, we adopted the approach presented in Mauss *et al*.^21^, which posits a model of emotions that conceptualizes them as experiential and physiological phenomena. According to this model, emotional responses are represented as dimensions rather than discrete states. As such, an emotional response encompasses both experiential and physiological aspects. Our study focused on recognizing emotions by analyzing physiological patterns, aligning with contemporary key theories in emotion research. These theories propose that physiological responses are fundamental components of emotional experiences^22^.

**Fig. 1:**
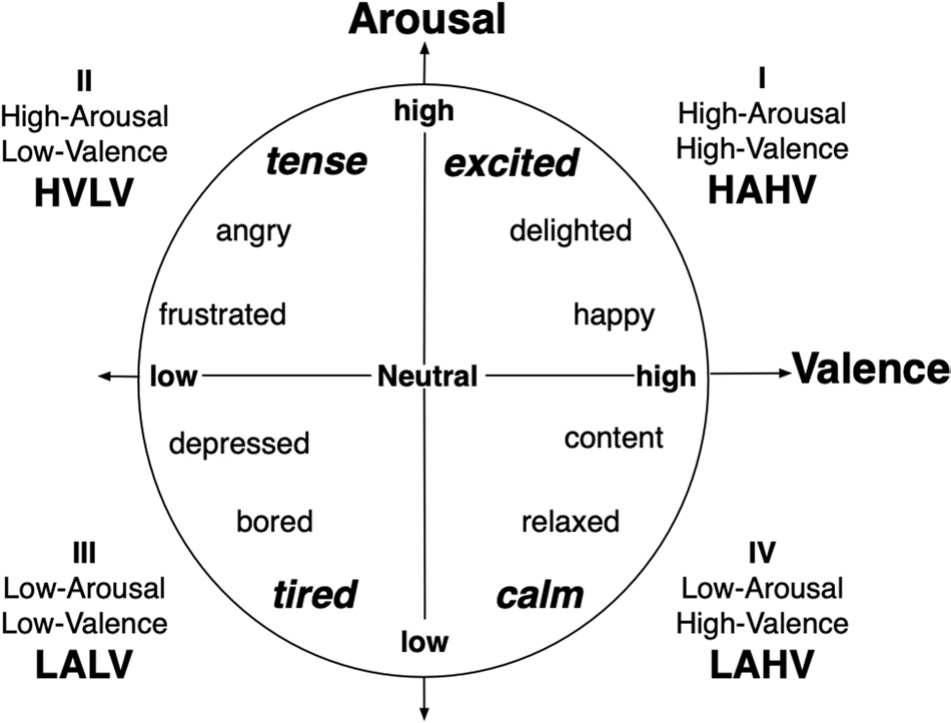
Illustration of a two-dimensional valence and arousal space. The five emotional states - calm, tired, tense, and excited - are highlighted in bold, plus the neutral. Each quadrant of the space, labeled with Roman numerals from I to IV, corresponds to the specific emotional set selected for investigation.

We adopted the circumplex model proposed by Russell^23^, where emotions are represented as continuous numerical values in multiple dimensions. The valence-arousal (VA) circumplex model (see Fig. 1), positions emotions along two orthogonal axes: valence and arousal. Valence indicates the degree of positive or negative feelings, while arousal represents the degree of excitement or calmness. To operationalize these dimensions, we defined a set of four emotions located at the extremes of each quadrant: High Valence & Low Arousal (HVLA), Low Valence & Low Arousal (LVLA), Low Valence & High Arousal (LVHA), and High Valence & High Arousal (HVHA), in addition to a neutral state at the center of the space.

### Experiment protocol

Two weeks before the experiment, participants were instructed to engage in mental imagery exercises to elicit the five emotions and a neutral state. Participants were encouraged to imagine various scenarios, such as film scenes, theatrical backdrops, personal memories, or any other events they believed could evoke each emotion under investigation.

**Fig. 2:**
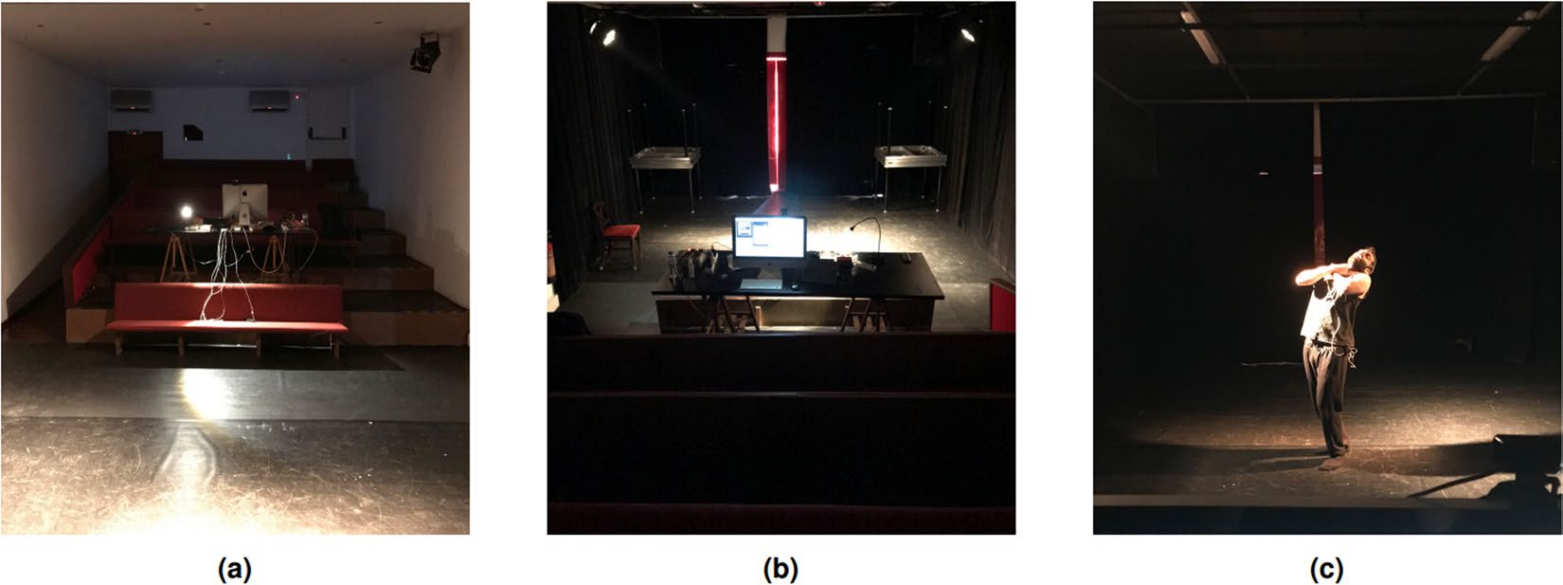
The experimental scenario, with participant’s consent for their likeness to be used. Panel (**a**) presents a stage perspective, depicting the space where the experiment was conducted, including the setup of the biosignal acquisition system. Panel (**b**) provides an audience perspective, illustrating the stage under controlled lighting conditions. Panel (**c**) offers an experiment perspective, showing a participant who has granted permission for the use of their image, eliciting one of the emotional states under study on the stage.

On the day of the experiment, participants received an oral description of the study procedures. Physiological sensors were attached to the participants, and they were seated comfortably on a chair positioned at the center of the stage (see Fig. 2a,b). The lighting intensity was dimmed to create an environment resembling a theater stage (please refer to Fig. 2c). A detailed explanation of the experimental protocol was then provided, outlining the sequence of emotions, the duration allocated for acting out each emotion, and the intermission period between emotions to allow for the re-establishment of a neutral state before proceeding to the next emotion. Before acting out each emotion, the name of the emotion was announced to ensure clarity and consistency across all participants. The emotional set was presented in the same order for each experimental session, and the entire experiment was approximately 40 minutes.

Following the experiment’s conclusion, the sensors were detached from the participants. Subsequently, we ran a feedback session wherein participants employed a touch-responsive application to provide their assessment. Within a custom application, each participant could recreate the emotional trajectory on a two-dimensional VA representation displayed on the screen. This interactive interface allowed participants to express their perception of emotional experiences by manipulating the positioning of points on the valence-arousal space. This feedback collection process aimed to gather valuable insights regarding the effectiveness of the research methodology. Participants were also encouraged to share any additional insights or observations related to the experiment. The experiment was repeated 15 days later to assess the temporal correlations between sessions.

### Data acquisition

The data recording in our study was performed using the BITalino Plugged kit^24^ in combination with an R-IoT device. The R-IoT device provided WiFi connectivity, enabling communication between the BITalino board and the computer via the User Datagram Protocol (UDP). When plugged onto the Universal Asynchronous Receiver/Transmitter of a BITalino (r)evolution device, the R-IoT streams single Open Sound Control^25^ packets containing all the exported data (IMU + BITalino). The BITalino (r)evolution Plugged kit was housed within a 3D-printed enclosure designed to safeguard its components.

For data acquisition and management, we developed custom software using the MaxMSP (https://cycling74.com/products/max) programming environment (see Fig. 4a). This software facilitated various functionalities, including listening to the BITalino hardware through the UDP port, parsing the data, visualizing the signals, formatting the data, and saving the physiological signals obtained from the board (see Fig. 4b). The software supported recording in two different formats: raw and resampled. The raw format involved logging the recordings at the sample rate of the board, i.e., 100 Hz, while the resampled format maintained a constant sample rate of two milliseconds, i.e., 500 HZ. The resampled version was an arbitrary selection based on the assumption that we could derive high-resolution interpolated versions for future analysis (see Fig. 4c). Both raw and resampled versions were automatically saved in files with unique identification for each recording session. Furthermore, the software facilitated the synchronized video and audio recording, ensuring that these components could be analyzed alongside the captured physiological data in future analyses.

**Fig. 3:**
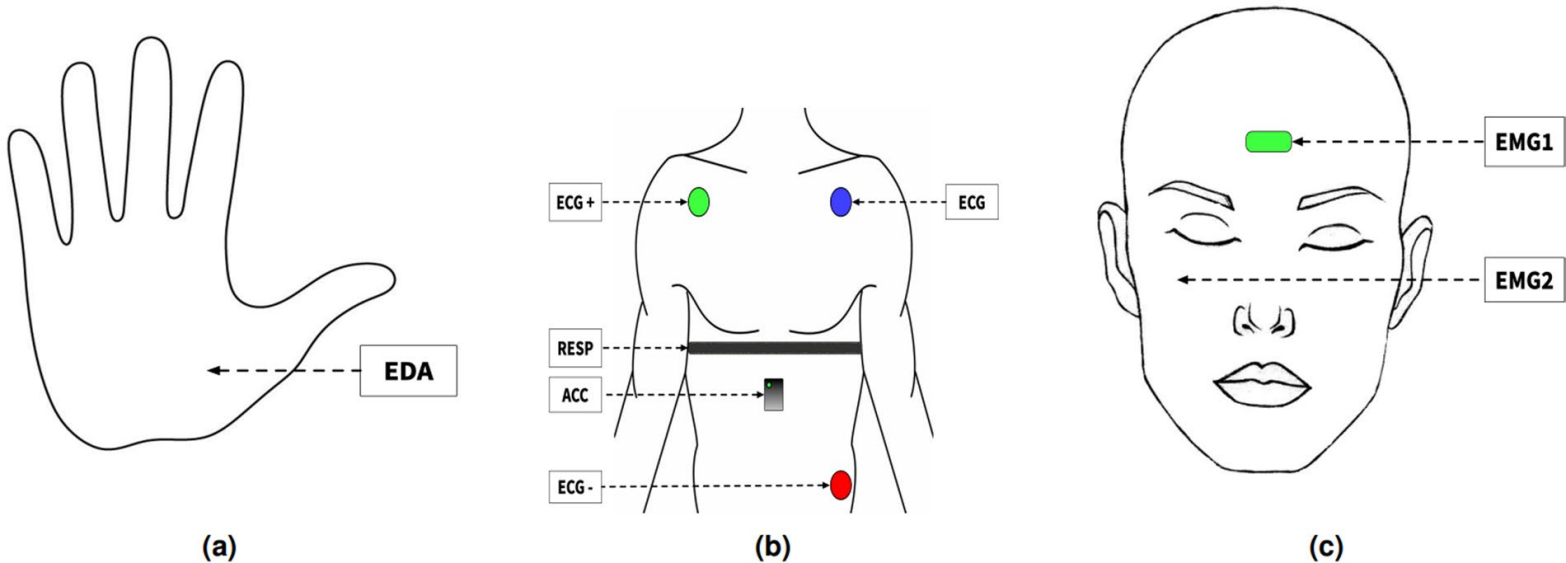
Sensor placement strategy adopted in the context of our study. Panel **(a)** showcases the placement of the EDA sensor in the non-dominant hand. Panel **(b)** points the location of ECG in Lead II configuration, the PZT in the thorax, and a three-axis digital accelerometer sensor. Finally, on panel **(c)** depicts the placement of the EMG 1 *corrugator supercilii* and EMG2 *zygomaticus major*.

**Fig. 4:**
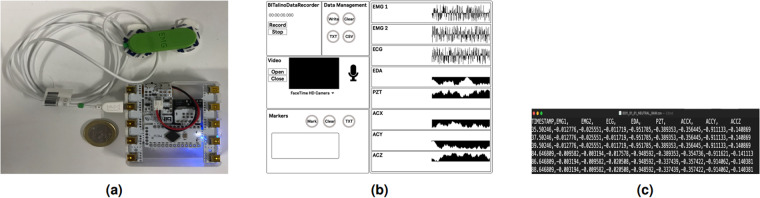
Illustration of the experiment instruments. Panel **(a)** showcases the placement of various sensors on different body parts of the participants. These sensors were connected to an (r)evolution Plugged kit board and a BITalino R-IoT device, which provided WiFi connectivity. To ensure protection and compatibility with theatrical performances, the entire setup was enclosed in a 3D-printed casing, as depicted in the image. A 1 euro coin is included in the image for size comparison. Panel **(b)** highlights the custom software developed for data recording and analysis. The software facilitated listening to the UDP port, parsing physiological signals, visualizing data, and saving data in raw and resampled formats. The image showcases the software interface utilized for data processing. Panel **(c)** emphasizes storing sensor data in a CSV file, where each participant and emotion is assigned a unique identifier. The CSV file was structured in column format, with the following data arrangement: timestamp, EMG1, EMG2, ECG, EDA, PZT, and acceleration data (X, Y, & Z axes).

Data was recorded using an Apple iMac with a 2.5 GHz Intel Core i5 processor, 20 GB DDR3 RAM, and a 21.5-inch screen. Video and audio recordings were captured using the iMac’s built-in microphone and HD camera.

### Assessment software

After each session, participants were presented with a video recording of their performance. Subsequently, utilizing a touch-responsive application, they were instructed to reproduce the emotional trajectory of each emotion on a two-dimensional valence- arousal (VA) space (see Fig. 1). This self-report assessment aimed to ascertain nuances in each emotion, the separation of emotions, and identify the gap between elicited emotion and the final result. Furthermore, participants were requested to provide a concise textual description of the mental imagery employed to evoke the emotions under investigation.

## Sensors & Instruments

Emotion recognition employing biomedical sensors is a well-consolidated research field relevant to AC and HCI communities. It has been explored in conjunction with other modalities^26,27^ and individually^28–30^. Although there is no report on the optimized placement position of a biomedical sensor setup^31^, we adopted the procedures depicted in Fig. 3. In the following paragraphs, we describe the physiological measures used in the present study. Finally, we provide the transformed sensor output values for each of the five physiological sensors used in the experiment.

The *electromyography sensor* (EMG) captures the electrical activity of surface or internal muscles, using noninvasive electrodes placed on the body surface or invasively in contact with muscle fibers^32^. In our study, these sensors were used for facial electromyography; sensors were positioned on the left side of the face, targeting the *corrugator supercilli* and *zygomaticus major* muscles, which display a higher mimicry rate in comparison to the right side^33^.

The *electrocardiography sensor* (ECG) measures the heart’s electrical activity using electrodes placed in contact with the body surface^34^. In our study, ECG electrodes were positioned according to the commonly used Lead II configuration^35^.

The *electrodermal activity sensor* (EDA), also known as galvanic skin resistance (GSR), captures changes in skin conductance resulting from activity in the sympathetic nervous system through electrodes applied to the palms of the hands or soles of the feet^36^. In our study, EDA was measured in the palm of the non-dominant hand^37^.

The *piezo-electric respiration sensor* (PZT) captures chest displacement induced by inhalation and exhalation activity using a piezoelectric element, a pressurized tube, or variations in inductance within a coil embedded in the fabric^38^. Respiratory rate was measured using an elastic chest belt adjusted in length to fit the participant’s thorax.

The *accelerometer sensor* (ACC) measures static or dynamic acceleration, typically employing a damped mass mounted on a spring^39^.

## Data Records

The DECEiVeR (**D**atas**E**t a**C**ting **E**mot**i**ons **V**alenc**e** a**R**ousal) dataset^19^ is publicly accessible via the Figshare open access repository. The entire dataset is consolidated into a single archive file, which can be accessed at (10.6084/m9.figshare.23579862).

For promoting different perspectives on the (**D**atas**E**t a**C**ting **E**mot**i**ons **V**alenc**e** a**R**ousal), the main DECEiVeR dataset^19^ structure is organized into five main folders: DECEiVeR_raw, DECEiVeR_resampled, DECEiVeR_session, DECEiVeR_arouval, and DECEiVeR_features. README files are available in each directory to explain the contents and organization of the data. Participant numbers are identified with the letters “ID” followed by a two-digit number (e.g., ID01, ID02), which is consistent across all DECEiVeR datasets. For example, the file “ID01_01_01_NEUTRAL_RAW.csv” corresponds to data from the first participant’s first session, during which they acted out the first emotion, namely neutral, in a raw format. The dataset is depicted in Fig. 6 and organized as follows:

**Fig. 5:**
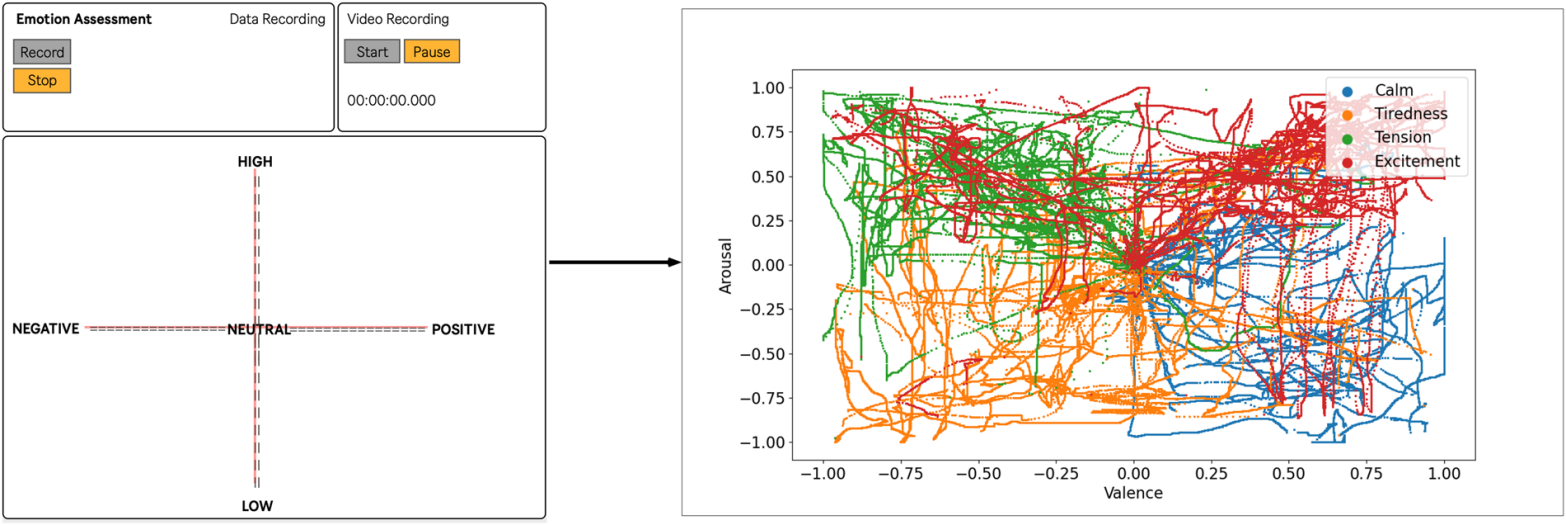
Participants’ self-assessment. The right part of the image illustrates the assessment software developed in our study. We prompted participants to reproduce the intended emotional trajectory while watching the video recording of their performance through a touch-responsive interface. The left part of the image depicts the self-reports and the obtained continuous annotations trajectories. The self-reports are in line with the emotions intended for each emotional category, i.e., each emotion category covers the designed ground-truth quadrant, with a distinction between different categories: Calm belongs to the HVLA; Tiredness to the LVLA; Tension to the LVHA; and Excitement to the HVHA, suggesting the participants successfully induced the expected degree of valence and arousal per emotion. We highlight the outliers in the valence dimension for Tiredness and Tension, and the arousal dimension in Tension and Excitement.

**Fig. 6:**
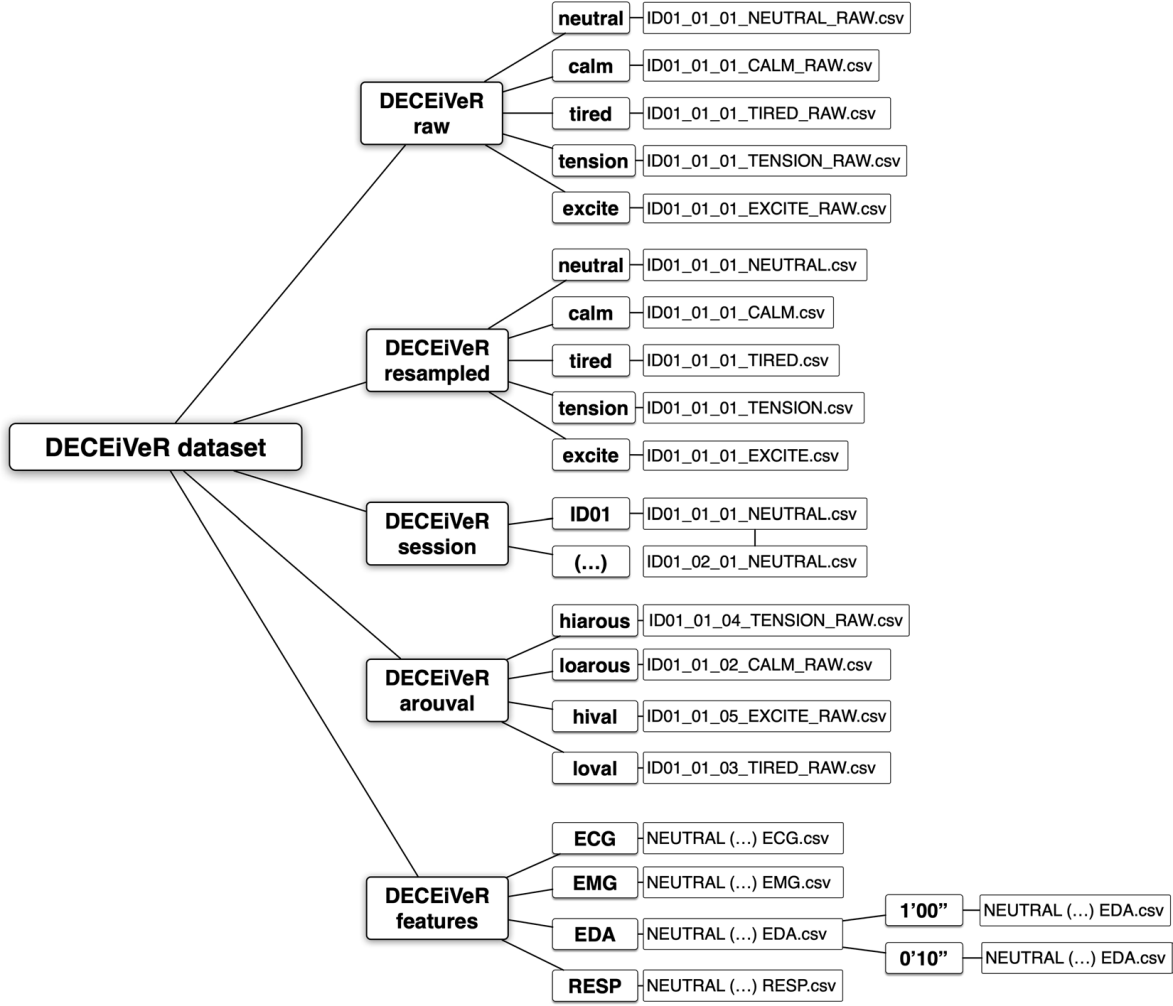
Tree diagram illustrating the directory structure of the DECEiVeR dataset^19^. The root directory, labeled [DECEiVeR dataset], comprises four main directories: [DECEiVeR raw], [DECEiVeR resampled], [DECEiVeR session], [DECEiVeR arouval], and [DECEiVeR features]. The [DECEiVeR raw] and [DECEiVeR resampled] directories include five sub-directories organized by emotions. Within each of these directories, individual files are provided for each participant in both sessions. The [DECEiVeR session] directory contains a sub-directory by participant containing individual files for each participant. The [DECEiVeR arouval] directory is structured according to emotional quadrants in each sub-directory, specifically high and low arousal and valence dimensions. Within each directory, individual files are available for each participant. Lastly, the [DECEiVeR features] directory is organized by sensor sub-directories containing individual files for each emotion. However, one exception is the [EDA] directory, which includes two sub-directories representing the two analysis windows adopted in our study: one for a ten-second window and another sub-directory for a one-minute window.

### DECEiVeR_raw

The [DECEiVeR_Raw] directory contains the raw data logs, representing the unprocessed data obtained from all 11 participants. The data is organized based on the corresponding emotion of each participant. We collected data at a sampling rate of approximately 100HZ. The [DECEiVeR_Raw] directory comprises approximately 556 megabytes (MB) of data. It is subdivided into five folders, each corresponding to one of the emotions investigated in the study. Each folder contains individual CSV files containing nine variables, each occupying its column.

- *Column 1*. Time in milliseconds of each acquisition sample
- *Columns 2-9*. Contain the transformed sensor output values sampled (values from −1 to 1) from the EMG1-*corrugator*, EMG2-*zygomaticus*, ECG, EDA, PZT, and three accelerometer axes.

### DECEiVeR_resampled

The [DECEiVeR_resampled] directory houses the resampled data logs from all 11 participants, referring to data adjusted to a sampling rate of 2 ms per sample. The *DECEiVeR_resampled* archive contains approximately 1.20 gigabytes (GB) of data. The directory is organized into five separate folders, each dedicated to one of the emotions examined in the study. Each CSV file within these folders encompasses nine variables, each occupying its own column.

- *Column 1*. Time in milliseconds of each acquisition sample at ∼7 ms per sample
- *Columns 2-9*. It contains the transformed sensor output values sampled (values from −1 to 1) from the EMG1-*corrugator*, EMG2-*zygomaticus*, ECG, EDA, PZT, and three accelerometer axes.

### DECEiVeR_session

The [DECEiVeR_Session] directory contains the resampled data for the 11 participants, arranged according to sessions one and two. The *DECEiVeR_Session* archive, comprising approximately 1.20 GB of data, is structured within a directory with 11 individual folders, each corresponding to a specific participant. These folders contain CSV files consisting of nine variables, each occupying its own column. This organization facilitates the analysis and exploration of the resampled data in a session-specific manner.

- *Column 1*. Time in milliseconds of each acquisition sampled at 500 Hz.
- *Columns 2-9*. Contains the transformed sensor output values sampled (values from −1 to 1) from the EMG1-*corrugator*, EMG2-*zygomaticus*, ECG, EDA, PZT, and three accelerometer axes.

### DECEiVeR_arouval

The resampled data from all 11 participants is stored within the directory, organized based on dimensions. It is further subdivided into four folders: [DECEiVeR_01_higharous] containing data logs related to tension and excitement, [DECEiVeR_02_lowarous] containing data logs related to calmness and fatigue, [DECEiVeR_03_posvalence] containing data logs related to calmness and excitement, and [DECEiVeR_04_negvalence] containing data logs related to fatigue and tension. Each file within these folders contains nine variables, each occupying its column. This directory structure allows for convenient access and analysis of the resampled data, enabling researchers to examine the dimensions and associated data logs separately.

- *Column 1*. Time in milliseconds of each acquisition sampled at 500 Hz
- *Columns 2-9*. Contains the transformed sensor output values sampled (values from 0 to 1) from the EMG1-*corrugator*, EMG2-*zygomaticus*, ECG, EDA, PZT, and three accelerometer axes.

### DECEiVeR_features

The directory encompasses the extracted features from the data logs of all participants, organized by individual sensors, specifically [01_ECG], [02_EMG], [03_EDA], and [04_PZT]. Extracting features from the raw signal allows each sample to be represented by a reduced set of values instead of thousands of variables. It is important to note that the [03_EDA] directory includes extracted features from both a 10-second and 60-second time window. The DECEiVeR_Session archive contains approximately 15 MB of data. This directory structure facilitates the analysis of extracted features from different sensors, providing researchers with a concise representation of the data while retaining relevant information for further exploration and interpretation.

## Data Pre-processing

In addition to the raw log files from the sensor data, we provide a statistical description and feature-based annotation of the biomedical sensor data. Feature extraction plays a crucial role in signal processing, reducing the data size while preserving relevant information. Higher-level analysis systems, such as emotion classifiers, often rely on this set of features to train their models. For feature extraction, we used BioSPPy^40^, a toolbox for biosignal processing written in Python, bundling together various signal processing and pattern recognition methods to analyze biosignals.

The statistical descriptors are categorized into temporal and spectral domains, calculated on a time window with 60-second time windows with 10-second overlaps^41,42^. This segmentation strategy enabled us to validate the collected data effectively. We extracted the most relevant features from each segment, as specified in Table 2, which presents the statistical descriptors, with each feature represented by a set of 11 descriptive statistics per analyzed window: minimum and maximum values, mean, difference, variance, root-mean-squared (RMS), standard deviation (SD), root-mean-squared standard deviation (RMSSD), kurtosis, skewness, and interquartile range (IQR). We report spectral descriptors features with ten spectral descriptors: total energy, spectral centroid, spectral skewness, spectral kurtosis, spectral slope, spectral decrease, spectral roll-off, and spectral variation. Table 3 presents the 18 extracted features organized by sensor type.

**Table 1:** Overview of the sensors used in the study.

Sensor	No.	Manufacturer	Model	Transformation equation	Units
EMG	02	BITalino	EMG 100716	$EM{G}_{1000mv}(v)=\frac{\left(\frac{ADC}{{2}^{n}}-\frac{1}{2}\right)\cdot 3,3v}{{1009}_{EMG}}$	*μC*
ECG	01	BITalino	ECG 100716	$EC{G}_{1000mv}(v)=\frac{\left(\frac{ADC}{{2}^{n}}-\frac{1}{2}\right)\cdot 3,3v}{{1100}_{ECG}}$	*mV*
EDA	01	BITalino	EDA 081217	$ED{A}_{1000mv}(v)=\frac{\frac{ADC}{{2}^{n}}\cdot 3,3}{0,132}$	*μS*
RESP	01	BITalino	PZT 280519	$PZ{T}_{ \% }=\left(\frac{ADC}{{2}^{n}}-\frac{1}{2}\right)\cdot 100 \%$	%
ACC (3 axis)	01	BITalino/RIoT	BR 20200207	—	{−8; + 8}g
GYR (3 axis)	01	BITalino	BR 20200207	—	{−2; + 2} °/s
ADC	06	BITalino	BR 20200207	—	—

The table includes (i) the type of sensor, (ii) the number of sensors used in the experiment, (iii) the manufacturer, (iv) the reference model, (v) any applicable conversion equations used to transform input values, and (vi) the physical units based on the International Standard units.

**Table 2:** Overview of the 19 statistical descriptors grouped into two domains: temporal and spectral.

Domain	Statistical descriptors
Temporal	Minimum and maximum value, mean, difference, variance, root-mean squared (RMS), standard deviation (SD), root-mean squared standard deviation (RMSSD), kurtosis, skewness and interquartile range (IQR)
Spectral	Total energy, spectral centroid, spectral skeweness, spectral kurtosis, spectral slope, spectral decrease, spectral roll-off, spectral variation

**Table 3:** Overview of the sensors used in the study and the corresponding 14 extracted features from the signals.

Sensor	Extracted features
ECG	Heart rate is the number of heartbeats per minute (HR min and HRmax)
	The time period between successive heartbeats (IBI)
	Interbeat intervals from which artifacts have been removed (NNi)
	Standard deviation (SD) of NN intervals (SDNN)
	Filtered data to remove noise (RR)
	Intervals that differ by more than 50 ms (RRi)
	Standard deviation of the average NN intervals for each 5 min segment (SDANN)
	Percentage of successive RR intervals that differ by more than 50 ms (pNN50)
	The number of adjacent NN intervals that differ from each other by more than 50 ms (NN50)
	Integral of the density of the RR interval histogram divided by its height (TRindex)
EDA	Tonic level of electrical conductivity of skin (SCL)
	Phasic change in electrical conductivity of skin (SCR)
	Phasic rate, rise and half-recovery
EMG zygomaticus	Statistical, temporal and spectral features (see Table 1)
EMG corrugator	Statistical, temporal and spectral features (see Table 1)
PZT	Statistical, temporal and spectral features (see Table 1)
	Respiration rate
	Interval of respiration peaks

## Technical Validation

We conducted a preliminary analysis to address three essential inquiries: (1) the efficacy of the *raw* physiological data in capturing and recognizing emotions under investigation; (2) the effectiveness of the *feature-based* physiological data in capturing and distinguishing emotions under examination; and (3) the temporal persistence of the physiological measurements between two-spaced sessions. We acquired data for the first two tests to determine which sensor yields the highest differentiation of emotions based on raw and feature-based data, respectively. The latter evaluation assessed the variability of physiological responses across the two sessions per participant and emotion, thereby shedding light on concerns about calibration and reproducibility. Ultimately, the findings aim to uncover the potential of physiological data in elucidating inner-elicited emotion. These results can provide valuable guidance for future endeavors in developing robust models for understanding and recognizing emotions evoked by internal stimuli.

**Fig. 7:**
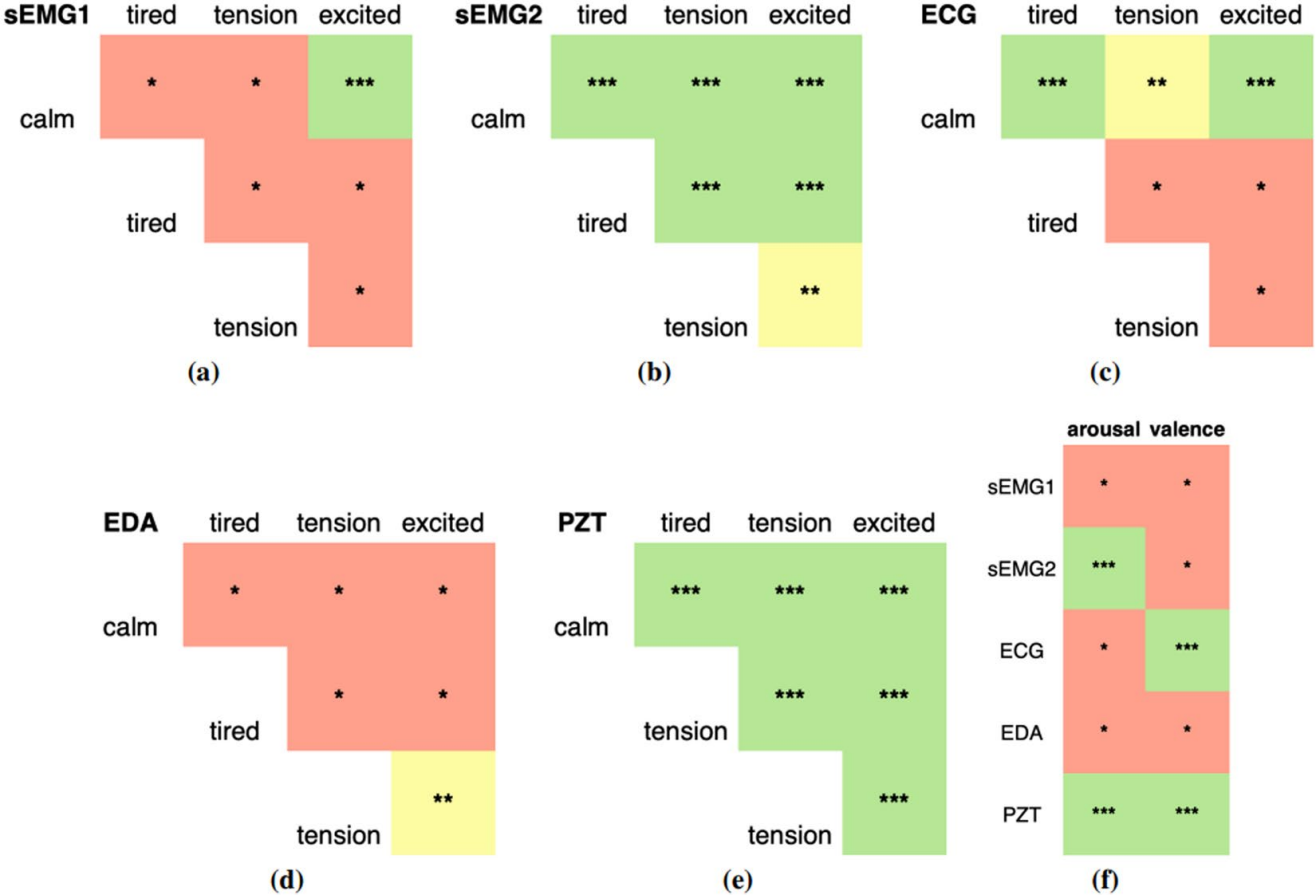
Significance levels from t-tests comparing the raw physiological data between emotions. The figure’s color scheme highlights low (red), moderate (yellow), and high (green) significance. Figures **(a)** and **(b)** refer to both EMGs. Figure **(c)** corresponds to the ECG sensor. Figure **(d)** corresponds to the EDA sensor. Figure **(e)** displays the results for the PZT sensor. Finally, Figure **(f)** depicts the outcome of the t-test analysis for an individual sensor according to the arousal and valence dimensions (please refer to Fig. 1). These figures visually depict the statistical significance of the t-test results, providing insights into the relationships between the sensors and emotions in the study.

To try to answer the first question, we focused on determining if there are significant statistical differences in the raw signal data between emotional pairs study^43^. To assess these differences, we applied t-tests on raw sensor data to compare the means between pairs of emotions and determine whether there was a significant distinction between them. The t-test provided us with a corresponding *p*-value, which indicates the strength of evidence against the null hypothesis. The *p*-value ranges from 0 to 1, with smaller values indicating stronger evidence against the null hypothesis. Researchers commonly establish threshold values to define the level of significance^44^. Commonly used thresholds include *p* ≤ 0.001, *p* ≤ 0.01 and *p* ≤ 0.05, denoting high, moderate and low significance. The applied test results are presented in Fig. 7 and illustrate the results, with green denoting a *p* ≤ 0.001, indicating highly significant differences. Yellow denotes a *p* ≤ 0.01, indicating moderate significant differences. Red denotes a *p* ≤ 0.05, denoting relatively lower but significant differences.

Results show that EMG2 and PZT differ best across all five emotional states. Please note that the placement of the EMG sensor suggests having a significant impact on recognizing acting emotions. The EMG1, placed at zygomaticus muscle activity, has the worst performance in differentiating the five emotions under study compared to the EMG2. While the PZT sensor results show better performance in capturing differences across both valence and arousal dimensions, the EMG2 and ECG show enhanced differentiation for arousal or valence, respectively.

**Fig. 8:**
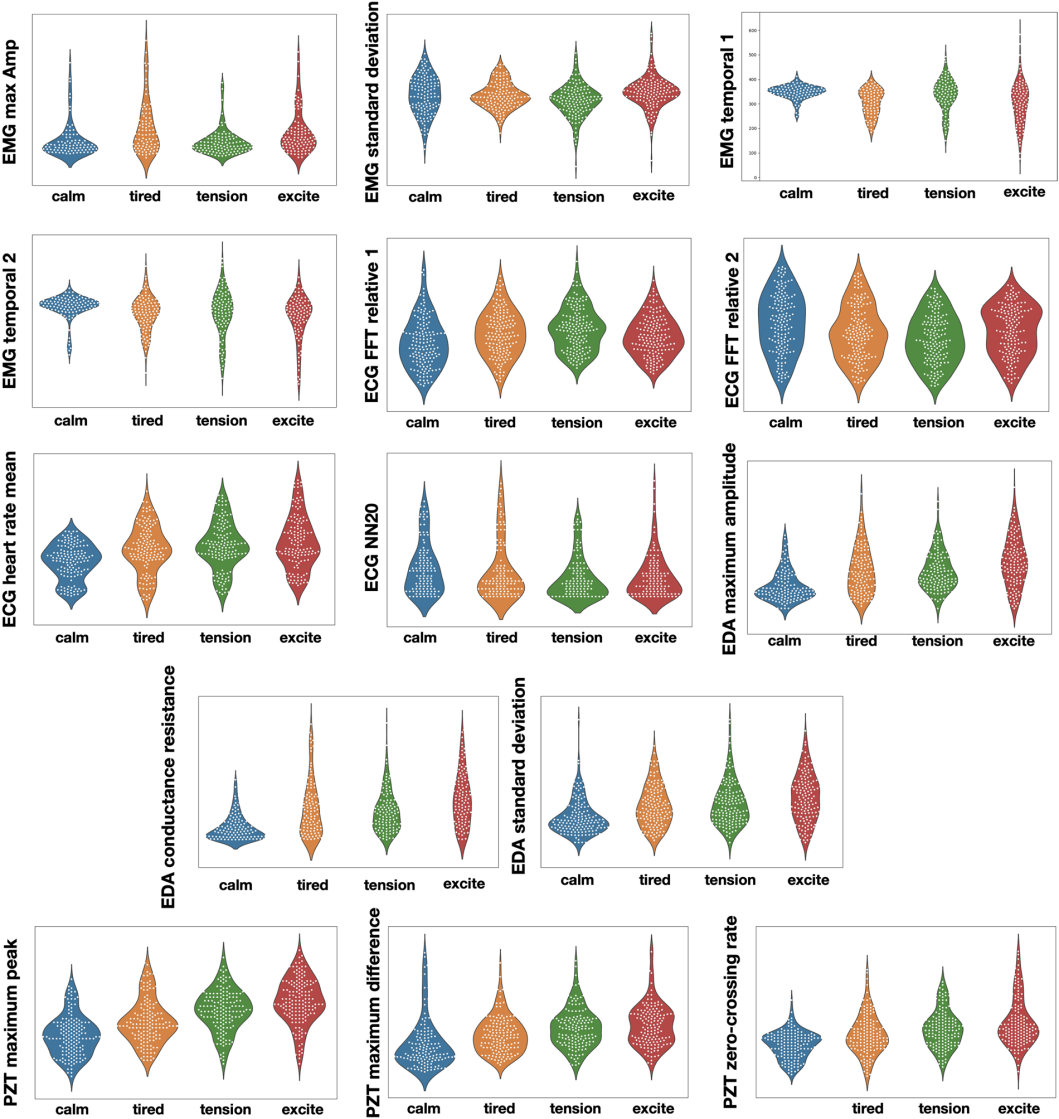
“Violin” plots of the distribution of the selected features values across different types of four emotions.

To investigate the effectiveness of the feature-based physiological data in capturing and distinguishing emotions under examination, we assessed which features from Tables 1 and 2 best differentiate the four emotions and plotted density and peaks in the data. The violin plots in Fig. 8 showcase the distributions of features commonly used for emotion recognition. Our preliminary analyses generally do not reveal a clear distinction between emotions, except for the three features extracted from the PZT sensor, which reveals better performance across the four emotions. The selected ECG features display a slight distinction between emotions. We could not identify clear patterns in the data for the remaining sensor set. The results show that physiologically-based features are characteristic of specific emotions. For instance, emotional excitement is associated with high SCR and elevated HR values, while feelings of calmness link to reduced respiration rates and low EMG1 activity.

The three tests mentioned above show findings worth examining. First, a calibration phase is crucial for adapting to the subject under analysis, especially when building classification models to understand complex emotional data. Secondly, the raw data’s feature selection is crucial to understand the dataset. The DECEiVeR features folder in our dataset contains a comprehensive set of features previously extracted from the raw signal for more in-depth investigation. Finally, a more subjective finding of our research is that expectations differ when inducing emotions through visual or auditory emotional content and asking the subject to elicit an emotion from his/her inner self. Moreover, suppose participants, like theatre actors, are highly skilled in acting emotions. In this context, they are expected to not deliver obvious and predictable responses, such as the non-trained population.

Finally, to examine the consistency of measurements across different sessions, we assessed the reliability of these measurements for each participant over time. In this context, ‘reliability’ pertains to the consistency of a specific measurement, particularly a physiological one, over a given period^45^. In our study, this period spanned fifteen days. The rationale behind conducting a repeat experiment after 15 days was primarily to understand the reliability of physiological measures in the context of acting performances rather than focusing exclusively on the long-term stability of the data.

In professional acting, the principle of repeatability holds critical significance. Actors need to be confident that a particular action or expression will reliably evoke similar emotional and physiological responses, especially in the dynamic environment of live performances. Our study was designed to investigate this aspect of repeatability, focusing specifically on physiological responses. Accordingly, we utilized reliability tests deemed most suitable for assessing the short-term consistency of actors’ emotional and physiological reactions. The results of these tests offer vital insights into the repeatability of emotional expressions in acting, a crucial component in the study of performance.

**Fig. 9:**
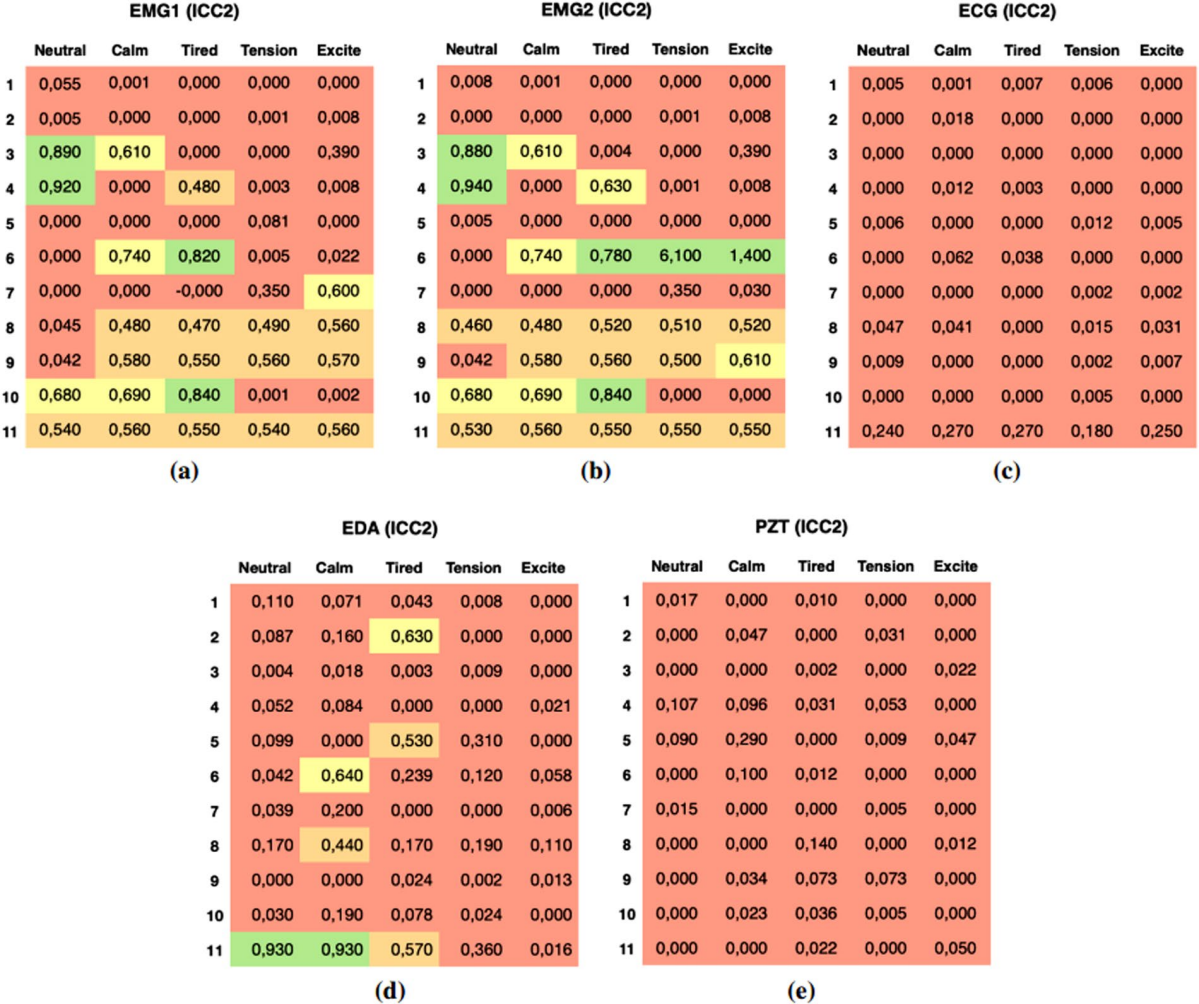
Correlation levels from ICC tests comparing data consistency through sessions. Panel **(a)** and **(b)** display the ICC reliability measurements for both EMG sensors, indicating the consistency and agreement of the measured muscular activity between sessions. Panel **(c)** illustrates the ICC reliability measurement for the ECG sensor. Panel **(d)** represents the ICC reliability measurement for the EDA sensor. Panel **(e)** showcases the ICC reliability measurement for the PZT sensor. Overall, the ICC reliability measurements provide valuable information about the consistency and stability of the physiological measurements across sessions, highlighting the reliability of the sensors in capturing and measuring specific physiological responses related to emotions.

We measured the reliability of measurements of four emotions from session 1 to session 2 with the same participants and measures. We employed the intraclass correlation coefficient (ICC), namely a twoway mixed-model with absolute-agreement metric^44^. The ICC is a standard index ranging from 0.0 to 1.0^46^. It provides guidelines for interpreting ICC levels, including the following thresholds: ICC ≥ 0.75 indicates “Excellent” reliability, ICC ≥ 0.60 and <0.75 indicates “Good” reliability, ICC ≥ 0.40 and <0.60 indicates “Fair” reliability, and ICC<0.40 indicates “Poor” reliability. The results are presented in Fig. 9, which illustrates the ICC values across all databases. EMG sensors are reliable across sections because they show a greater number of ICC values ≥ 0.75 The performance of the HIGH ICC set (EMG1 and EMG2) was found to be medium-high, indicating good reliability. However, the performance of the LOW ICC set (ECG and PZT) was low in reliability. This observation can be attributed to the participants’ explicit control over muscular movements, as measured by EMGs, compared to their limited control over the autonomic nervous system, as measured by ECG and EDA sensors.

Future work in this research field should further explore the reliability of measurements by testing different types of ICCs. By assessing the data using various ICCs, researchers can determine if the reliability results differ significantly, providing valuable insights into the robustness of the measurements. Additionally, extracting meaningful features from the collected data is essential for future investigation. Researchers can develop more informative and efficient emotion recognition and analysis models by identifying relevant physiological patterns and characteristics. Feature extraction techniques can help uncover hidden patterns in the data and provide valuable insights into the physiological underpinnings of emotions.

In the context of artistic studies (the application scenario of our study), the conducted statistical analysis and results offer guidelines that can play a pivotal role in enlightening creatives as they design their theatrical performances. For instance, understanding the best typology of sensors associated with specific emotions (or emotional dimensions) can provide invaluable insights for artists aiming to evoke emotional responses beyond classification. Furthermore, assessing sensor response consistency over time for each emotion is critical. It allows artists to gauge the reliability of their chosen sensor in conveying intended responses. Current evaluation provides valuable directions for art directors to ensure some degree of consistency across multiple performances, thus contributing to the enduring quality of the artistic work.

Lastly, future research should experiment with machine learning models to develop robust and accurate emotion recognition systems. By training and evaluating various machine learning algorithms on the collected physiological data, researchers can explore the potential of these models in automatically detecting and classifying emotional states. This approach can contribute to developing real-time emotion recognition systems with applications in affective computing, human-computer interaction, and virtual reality.

## Data Availability

The code used for the experiment is publicly available at platform and cloud-based service for software development and version control GitHub. https://github.com/DECEIVER-dot/BITalino-Toolbox. We developed the code for these applications in MaxMSP. All required packages are listed in the requirements.txt file. This repository contains three applications developed in MaxMSP that enable direct communication with an IRCAM R-IoT module embedded in a BITalino board. The applications provide various functionalities, including biosignal data recording, Bluetooth connectivity, and interactive annotation while viewing video recordings of experiments. Please note that the software is designed to work with the MaxMSP programming environment and requires external libraries for specific functionalities. The first application allows seamless communication via a USB connection with the IRCAM R-IoT module on the BITalino board. It lets the user record biosignal data directly into a CSV file format with two different sample rates. The recorded data is saved in its raw form without normalization or interpolation. Please be aware that the current software version does not include data normalization or interpolation. The second application builds upon the functionality of the first application but adds Bluetooth connectivity as an alternative communication method. With this application, the user can connect the IRCAM R-IoT module on the BITalino board and the computer using Bluetooth. It provides the same data recording capabilities as the USB version, but motion data is not recorded with this application version. Experiment Annotation with Video Recording (Mira and iOS) The third application is designed for interactive annotation while viewing video recordings of experiments. Developed in MaxMSP, it requires the installation of external libraries for the Mira interface, which provides enhanced interaction capabilities. Additionally, this application relies on an iOS device to run the software effectively. Using this application, researchers or experimenters can annotate the video recordings in real-time, allowing for precise and synchronized annotation of events or observations. We implemented code in Python using the BioSPPy library to extract relevant information from biosignals. BioSPPy is a Python library for biosignal processing providing a set of algorithms for processing and analyzing physiological signals, such as electrocardiography, electrodermal activity, and electromyography, to name a few. BioSPPy simplifies extracting relevant information from biosignals and enables researchers and developers to focus on their analysis tasks. BioSPPy offers various modules and functionalities, including signal processing and feature Extraction such as HRV, EDA, and EMG analysis. BioSPPy is an open-source library and can be easily installed using Python package managers like pip or conda. Its modular design and user-friendly API make it accessible to beginners and experienced biosignal processing researchers. For more information, documentation, and code examples, please visit the official BioSPPy GitHub repository at (https://github.com/PIA-Group/BioSPPy). RStudio (https://posit.co/download/rstudio-desktop/) code was developed for conducting tests and interclass correlation analysis. The provided code allows users to perform various statistical tests and calculate interclass correlation coefficients using R programming language. Please note that the file naming and location should be adjusted according to the user’s computer file structure. We include the required external libraries for running the tests.
